# Uninterrupted Development of Two Aphid Species Belonging to *Cinara* Genus during Winter Diapause

**DOI:** 10.3390/insects11030150

**Published:** 2020-02-28

**Authors:** Roma Durak, Jan Dampc, Jagoda Dampc, Sławomir Bartoszewski, Anna Michalik

**Affiliations:** 1 Department of Experimental Biology and Chemistry, University of Rzeszów, Pigonia 1, 35-310 Rzeszów, Poland; jdampc@ur.edu.pl; 2 Department of Plant Physiology and Ecology, University of Rzeszów, Rejtana 16c, 35-959 Rzeszów, Poland; jadampc@ur.edu.pl; 3 Department of Biochemistry and Cell Biology, University of Rzeszów, Zelwerowicza 4, 35-601 Rzeszów, Poland; slawek@univ.rzeszow.pl; 4 Department of Developmental Biology and Morphology of Invertebrates, Institute of Zoology and Biomedical Research, Jagiellonian University, Gronostajowa 9, 30-387 Kraków, Poland; a.michalik@uj.edu.pl

**Keywords:** aphids, diapause, development, overwintering

## Abstract

Aphids are herbivores carrying the status of major pests for crops and ornamental plants. The many specific biological features of aphids allow them to survive unfavorable environmental conditions. As for other insects, a predominant strategy for aphids surviving winter, is laying diapausing eggs. During diapause, the expression of development may vary between species. Most of the insects stop growing during diapause; however, there is limited information about this process. We immunostained the embryos of aphids in order to detect cell division during diapause. Here, for the first time, we present that two species of aphids belonging to *Cinara* grow and develop throughout the duration of the winter diapause. Our results showed that the “resting stage” does not occur in embryos of these aphid species. The embryo of *C. cupressi* and *C. juniperi* undergoes a type of diapause, with slow growth. It seems that this feature is conducive to the rapid development of embryos in the egg, which may be another specific feature for aphid biology of overwintering.

## 1. Introduction

Aphids (Hemiptera, Aphidoidea) are herbivores carrying the status of major pests for crops and ornamental plants. A high number of generations during the vegetation season, high fertility of females, and telescoping generations that allow shortening the development and simultaneous existence of many generations in the environment, together with parthenogenetic reproduction, contribute to the spread of particular aphid species onto many host plants. Aphids, as very flexible insects, very quickly respond to changes in environmental conditions [1]. Long-term environmental changes result in changes in migration periods, changes in species’ diversity and ranges of species when seasonal changes could have influence on period colonization of host plants, number of generations per year, or fertility. [2,3,4,5,6,7,8]. Many specific biological features of aphids allow them to survive unfavorable environmental conditions.

Diapause and quiescence form two different types of dormancies in insects. The quiescence is dormancy caused by unpredictable, irregular stress, for example, acyclic environmental changes such as high or low temperatures, lack of food, or drought. In this case, the response of the insect should be quick and immediate. Diapause has been selected by regularly occurring seasonal fluctuations in temperature, humidity, or food. This type of stress is predictable so the insect can prepare for it sooner, through physiological and behavioral changes. It means that diapause starts before the environmental stress, when all conditions permit development. The process of diapause is induced by the photoperiod and the gradual decrease in temperature [9]. Diapause programming involves the capture of photoperiod information by the central nervous system of females, followed by a cascade of biochemical reactions that culminate in the transfer of a molecular regulator that promotes a dormancy state in embryos [9]. This mechanism takes place when diapause induction occurs in the maternal generation and diapause is initiated in the following generation. This is the case of aphids and species with diapausing eggs. According to Tauber et al. [10], this is the dynamic state of the low metabolic activity process that is genetically determined and is under hormonal control, contrary to quiescence. Hodek [11] indicates that during diapause, morphogenesis is arrested, but growth, mobility or feeding are not stopped, in some insects. One of the hallmarks of insect diapause is cell cycle arrest in target tissues [12]. Distinguishing between these two types of dormancy is important, and some studies indicate that eggs of some insects, such as mosquitoes, can undergo diapause or/and quiescence [9]. According to Beck (1962) [13], diapause is the state of arrested development in which the arrest is enforced by a physiological mechanism rather than by unfavorable environmental conditions. A “resting stage” is a widely reported characteristic of diapause during which occurs a reduction or elimination of cell division and a cessation of morphological development [10,14,15,16]. The diapause process is an important chance for insects to survive seasonal changes in the environment, both in summer (aestivation) or winter (hibernation). Depending on the species, diapause could involve different developmental stages of insect: eggs, larval, pupas, or adults. Most studies focused on the diapause process of holometabolous insects and the characteristic stages such as larval or pupal diapauses [11,16]. In particular, *Drosophila melanogaster* is cited as a model organism to study the genetic base of expression diapauses across latitudes [17,18].

With insects, a predominant strategy for surviving winter, is egg-laying [19,20,21]. There are a number of environmental factors that can play a central role in completion of diapauses, such as temperature, photoperiod, light, and water. Those aphids that develop partenogenetically during spring and summer also produce eggs at the end of the vegetation season (holocyclic aphids). The key stimuli for the aphids to produce eggs are reduced photoperiod and lowering temperatures in autumn [22], as only a small number of species of aphids survive the winter as active forms [23]. This survival strategy is typical for anholocyclic aphids, which continues parthenogenetic reproduction all the way through the year. The laying of eggs is the most beneficial and effectual means of winter survival in temperate regions—especially as eggs can survive up to −40 °C [24]. It was widely believed that aphid eggs overwinter in obligatory diapause, not quiescence [16,25,26,27]. Aphid eggs undergo dormancy seasonally, so aphids are preparing for diapause in August and eggs are laid from October, before low temperatures occur. Diapause of aphids lasts a certain time, and the appearance of favorable environmental conditions (comfortable ambient temperature) does not end it [22,25,27]. 

During diapause, the expression of development may vary between species. Insects usually stop feeding and growing during diapause, but some diapausing embryos of representatives of the Orthoptera order may go through morphometric development [28]. The main work about aphid diapause was carried out by Behrendt [25], where he concluded that diapause periods involve developmental arrest and during this period, aphids are resistant to environmental stressors, especially temperature. This paper presents three phases in egg diapause of *Aphis fabae*: pro-, meso-, and metadiapause. When the eggs enter the embryonic development diapause, they slow down and the current development stages are prolonged [26]. The intensity of diapause is defined by the duration of the period independent of temperature—mesodiapause [25]. Until recently, diapause was noted for the majority of embryos of *Schizaphis graminum* (Rondani, 1852) and *Aphis fabae* (Scopoli, 1763) [25,27,29]. Several papers provided additional information regarding diapause termination and hatching of aphid species *Rhopalosiphum padi* (Linnaeus, 1758) and *Aphis spiraecola* (Patch, 1914) [30,31]. The research on diapause process of *A. pisum* indicated that early stages of embriogenesis progressed at a mesodiapause phase [32]. These authors showed that morphological development progressed slowly, but continuously, throughout embryogenesis of *A. pisum*. It suggested that the embryo in the egg does not experience complete developmental arrest [32]. 

Embryogenesis is a process by which a multicellular organism develops from a single cell. The process of embryogenesis begins after fertilization when genetic material from oocyte and sperm fuse to form a zygote. The first step of embryogenesis is a cleavage, during which a series of mitotic divisions of zygote take place and blastula is created. After cleavage blastula undergoes a gastrulation process, as a result, the primary germ layers (endoderm, mesoderm, and ectoderm) are formed. The last stage of embryogenesis is organogenesis, i.e., the formation of organs. Like in the other Hemimetabola, the developing germ band becomes totally immersed in the yolk mass, and undergoes a series of complex movements (blastokinesis). Two stages of blastokinesis are distinguished: anatrepsis, when the fully segmented embryo lies immersed in the yolk; and katatrepsis, when the embryo turns to adopt its final orientation with the head directed toward the anterior pole of the egg [27].

*Cinara (Cupressobium) cupressi* (Buckton, 1881) and *Cinara (Cupressobium) juniperi* (De Geer, 1773) are a holocyclic species of aphids, feeding on plants from the Cupressaceae family, the first mainly *Cupressus lusitanica, Cupressus macrocarpa, C. sempervirens, Chamaecyparis lawsoniana, Thuja occidentalis, and Juniperus* sp. and the second species are connected with *Juniperus* sp. The life cycle of *C. cupressi* consists of nine partenogenetic generations and a sexual generation. The average life span of the aphid generations range from 30 days to 50 days. Alate males were observed in the middle of October and after copulation, the amphigonic females laid from two to six eggs, beginning in the middle of October [33,34]. The life cycle of juniper aphid consisted of eight parthenogenetic generations and the sexual generation of *C. juniperi* occurs in September and October [35]. As the cypress aphid and juniper aphid are considered to be serious pests of ornamental plants, and the eggs of these species are rather big, these are the best model for examining the embryonic structures and process of diapauses. The cypress aphid is also considered to be one of the world’s 100 worst invasive alien species. Here, for the first time, we present the example of two species belonging to the *Cinara* genus, with embryonic development taking place in the natural conditions of a temperate climate, in which morphological development of embryos occurs during the “resting stage”, suggesting that this is not, as previously thought, an obligatory phase of an embryo’s development.

## 2. Materials and Methods 

### 2.1. Aphids and Egg Material

The aphids *Cinara cupressi* and *Cinara juniperi* were bred in a prepared habitat on *Juniperus sp*. at the University of Rzeszow (Poland) in natural conditions. We monitored subsequent generations of parthenogenetic females during the summer. Aphids were kept on plant branches in separate clip cages. Each clip cage contained 20 aphids. In Autumn, at temperatures 13–15 °C and a shortened photoperiod 10:14 LD, we obtained males and oviparous females, which were also bred in separate clip cages. After mating, the females laid their eggs; the eggs used for the experiment came from different females. The date of laying was monitored; the cohort of eggs were at the same ages, and the eggs remained on the plants throughout the experiment. The early stages of embryogenesis of the aphid, up to 15th day of development, were described by Miura et al. (2003) [36]. During prodiapause, we observed a change in the egg’s color from yellow to black (due to the deposition of serosal cuticle), and in the next stage a change in the position of the bacterial mass (bacteriocyte). The bacterial mass moved from the posterior end of the egg towards the center, and stopped migrating at one third of the length of the egg near the posterior end of the embryo. According to Shingleton et al. (2003) [32] and our findings, this moment is the time when the embryos enter diapause. By the end of this period, the embryo has completed anatrepsis, the first stage of blastokinesis, when the embryo is fully segmented with visible leg buds and lying immersed in the yolk. The embryos of aphids enter diapause at the end of anatrepsis [25]. The moment of diapause cessation is followed by katatrepsis, the second stage of blastokinesis [32]. In this time, the embryo membranes fuse posteriorly and break, the amniotic cavity opens and the embryo turns to adopt its final orientation with the head directed toward the anterior pole of the egg [27]. The embryos started to grow rapidly after katatrepsis and the last stages of organ differentiation took place, where embryonic cuticle and egg burster were observed.

From day 16, we started collecting samples every 7 days. The eggs were left on the host plants during winter time so that we could analyze their development. In each time point, we took 20 eggs from the cohort and dissected it every 7 days. We took the samples from day 16 to day 105, and the experiments were run for two years during the winter seasons from October to February in 2016/2017 and 2017/2018.

### 2.2. Fixing the Embryos, Dissection, and Staining

Eggs were placed in 50% bleach for 2 minutes to be dechorionated. They were then placed in a scintilation vial that contained 4% formaldehyde in PBS: heptane (1:1). The glass tube was shaken for 30 minutes at room temperature. Formaldehyde was replaced with methanol and the tube was shaken until the vitelline membrane was burst. The embryos were washed in methanol several times. The next step was to remove the embryo shields. To this end, the eggs were glued to the microscope slide with double-sided sticky tape and covered with PBS. The embryos were cleaned with preparative needles and transferred back to PBS. The embryos were transferred to methanol and stored at −20 °C. 

In order to detect cell division during diapause, we immunostained the embryos with Anti-phospho (Ser10)-acetyl (Lys14)-Histone H3 rabbit polyclonal antibody that detects Histone 3 phosphorylated at serine 10 and acetylated at lysine 14. The fixed biological material was washed once in ethanol for 3 minutes, then the medium was changed to PBS and agitated for 3 minutes. The last three washes were in 0.1% Triton-X 100 in PBS (PBT) and lasted 3 minutes each. The next step was to block non-specifically protein binding sites, the process lasted 30 minutes, and it was carried out in PBT with the addition of BSA (1% BSA in PBT). The BSA was removed and the primary antibody (Millipore) was used at a dilution of 1:5000. The whole was agitated for 2 hours at 25 °C. The whole was then washed three times in PBT, 5 minutes each time. A secondary antibody (Alexa Fluor 647; Invitrogen, Carlsbad, CA, USA) was added at a dilution of 1:1000, agitated for 2 hours at 25 °C. The samples were then agitated three times 5 minutes in PBT. After the last wash, DAPI was added and incubated for 10 minutes. The preparation was made (PBS in 90% glycerol with 0.02% azide).

### 2.3. Embryo Size Measurement

The size of aphid embryos was determined using the Microscope Software AxioVision LE based on photos taken with a confocal microscope (Zeiss LSM710, Carl Zeiss Microscopy GmbH, Jena, Germany). The total length of the embryo and the length of the third leg were measured. Where the embryo was distorted, the length was estimated as a sum of several linear measurements.

### 2.4. Statistical Analysis

All data are reported as means ± SD, n = 20 for each season, where each replication represents an individual aphid. We presented a mean length of body and leg on the base data for two winter seasons. We used one-way ANOVA and Duncan’s multiple-range tests to show the difference between samples collected in time, with significance level *p* ≤ 0.05. All statistical analyses were conducted using Statistica ver.10.

## 3. Results

**Figure 1:**
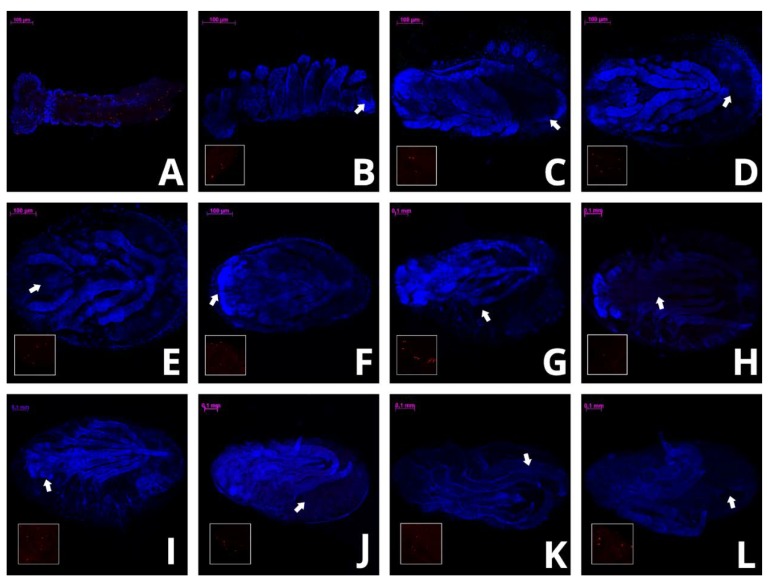
Development of *Cinara cupressi* embryo; in the gap there are red color cells in mitotic process from the place pointed by the arrow. The pictures show the stages of development on particular days of embryogenesis: (**A**) 16, (**B**) 28, (**C**) 35, (**D**) 49, (**E**) 56, (**F**) 63, (**G**) 70, (**H**) 77, (**I**) 84, (**J**) 91, (**K**) 98, (**L**) 105. All scale bars are 100 μm long. The embryos have a head directed to the left side.

**Figure 2:**
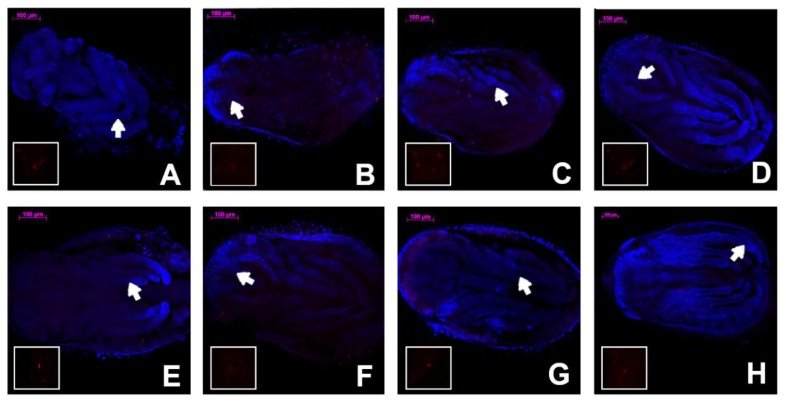
Development of *Cinara juniperi* embryo; in the gap there are red color cells in mitotic process from the place pointed by the arrow. The pictures show the stages of development on particular days of embryogenesis: (**A**) 16, (**B**) 21, (**C**) 35, (**D**) 42, (**E**) 49, (**F**) 56, (**G**) 63, (**H**) 70. All scale bars are 100 μm long. The embryos have a head directed to the left side.

**Figure 3:**
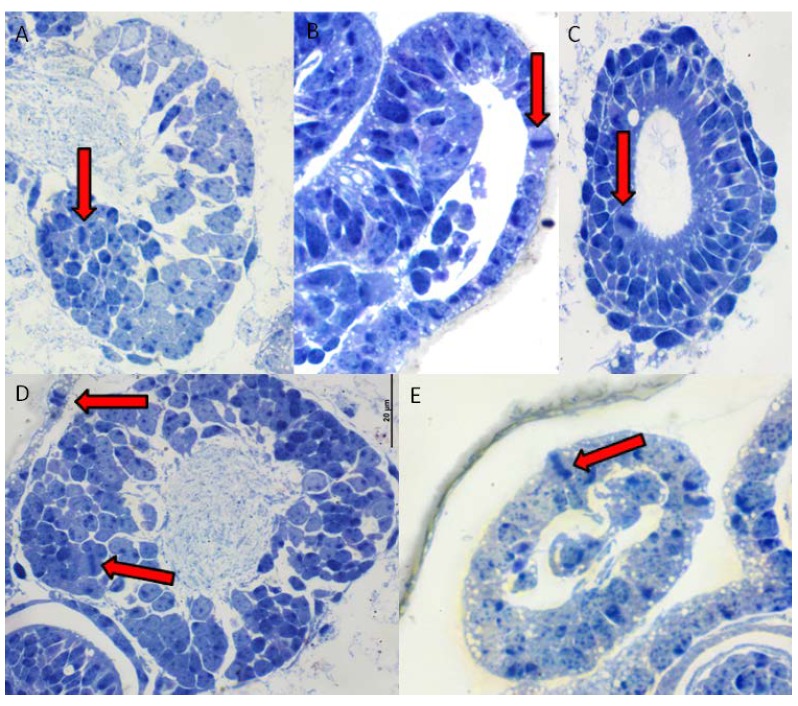
The mitotic cells divisions (pointed by the arrows) in *C. cupressi* aphid’s body: (**A**) nervous system, (**B**) body, (**C**) body, (**D**) nervous system, (**E**) leg. All scale bars are 20 μm long.

**Figure 4:**
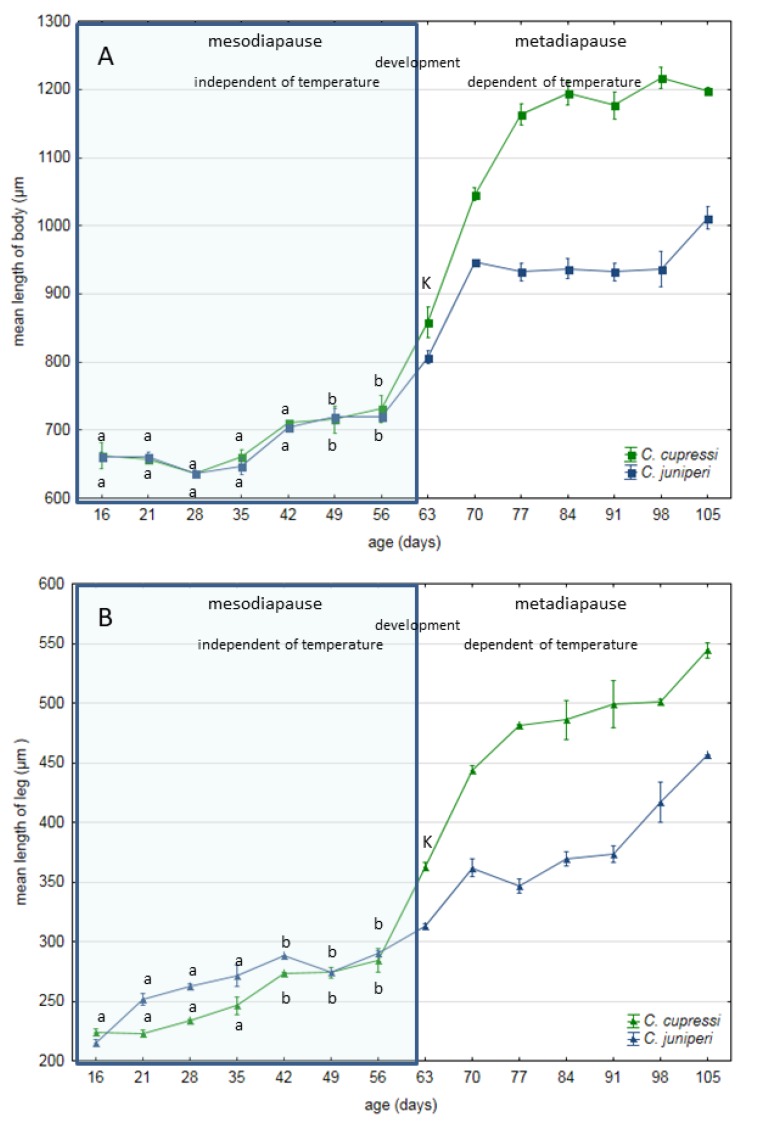
Changes in mean body length (**A**) and third leg (**B**) of aphid embryos during the diapause process in two winter seasons, 2016/2017 and 2017/2018. Statistical differences in mesodiapause phase were marked with different letters K – katatrepsis stage.

**Figure 5:**
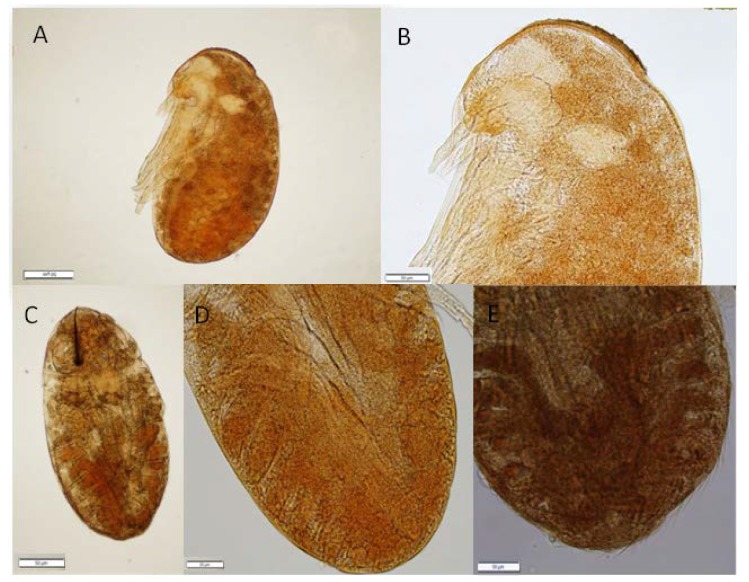
The process of creating a cuticle, egg burster and first hairs of *C. cupressi* (**A**,**B**,**D**) 77 day of development, (**C**,**E**) 84 day of development. All scale bars are 50 μm long.

On the 16th day of development, embryos of *Cinara cupressi* and *Cinara juniperi* had completed segmentation (Figure 1A and Figure 2A). From this time to the end of our experiments, we observed mitotic cells divisions (Figure 1 and Figure 2). The mitotic divisions were especially frequent in the 16 and 21 day body of aphids (Figure 1A and Figure 2B). The embryos from the 28th day were characterized by a large presence of cells in the mitosis phase, but mainly located in the legs (Figure 1B and Figure 3). The use of the anti-Histone H3 allowed observation of cell divisions in all examined embryos. The embryos in the following days were characterized by numerous mitoses that were visible throughout the entire body (Figure 1C,D and Figure 2C–E). Body length of the embryos increased steadily and from the 49th day the differences in body length compared to the 16th day were statistically significant (Duncan’s test, *p* ≤ 0.05, Figure 4). Starting from the 56th day of development, the embryos rapidly increased their body length (Figure 4). In the next stage, between 63 and 70 days, the embryos underwent katatrepsis (Figure 1F,G and Figure 2G,H). In this stage, the body of embryos had cells with visible mitotic divisions and the body length was significantly and statistically different from previous stages (Figure 4). The average length of the leg also increased regularly, and from the 42nd day of development until the 70th day, it was statistically different from the length of the embryo’s legs on the 16th day (Duncan’s test, *p* ≤ 0.001) (Figure 4). After katatrepsis, the average length of the body was almost twice as large as the embryos on the 16th day of development (Figure 1H and Figure 2H, Figure 4). The number of dividing cells began to decrease from the 77th day of development, but in each of the embryos mitosis was observed both in the body and the legs (Figure 1H–L). From day 77, a thin cuticle on the body of the embryos was observed. At the front end of the body, a delicate but well-visible egg burster was observed, which later became stronger in the following days. On day 84, the embryos of *C. cupressi* and *C. juniperi* were covered with an embryonic cuticle (Figure 1I). The egg burster was visible on the anterior end of body (Figure 5A–C). The average width of egg burster on the 77th day of development was 7.59 µm, while on day 88 it was 15.16 µm. We observed that the body of the embryos of *Cinara* was covered by short hairs from day 84 (Figure 5D,E). From the 77th day of development to the 105th day, the body length of the embryos only increased a little and it did not differ significantly from the subsequent stages of development. A similar result was obtained with respect to the average leg length, because there were no statistically significant differences between the length of the embryonic legs at day 77 of development and on the following days (Figure 4). Based on the conducted experiment, it was found that the *Cinara* embryos are characterized by constant development during the diapause phase and did not rest during the winter months. Cell division results in continuous, regular growth of embryos, initially mainly in the legs, but also in the entire body. The average length of the body of embryos after katatrepsis is over 1.7 times longer than on the 16th day of development, and the average length of the legs is more than twice as long. To the beginning of katatrepsis, the average increase in length of the body of *C. cupressi* was about 29%, and for *C juniperi* about 21%. The average increase in length of the legs of *C. cupressi* and *C. juniperi* was about 62% and 40%, respectively. After katatrepsis, the average increase in length of the body of *C. cupressi* was about 40%, and for *C. juniperi* about 25%. The average length of leg increased by approximately 50% for *C. cupressi,* and 45% for *C. juniperi*. 

## 4. Discussion

Our results showed that diapause in examined species of *Cinara* does not have a resting stage, but the embryos are constantly growing and developing between the anatrepsis and katatrepsis period. The only information about the diapause process of *Acyrthosiphon pisum* (Harris, 1776) indicates that it was a slowing process of growing [32]. However, successive stages did not differ significantly between each other. In the case of the *Cinara* aphids, the diapause process was characterized by substantial growth. We observed that the subsequent stages differed statistically from each other, and the embryos that passed katatrepsis were about one and a half times larger than the initial stage. Similarly to *A. pisum*, the growth of mainly the legs was observed initially, but the length of the body also increased from the 16th to 70th day of development. The period of embryo development up to the katatrepsis period is resistance for environmental influence and it is independent of ambient temperature [32]. *Cinara* embryos developed progressively until the end of katatrepsis, which means throughout the diapausa period. In naturally developing eggs in the winter, katatrepsis and the end of diapause were observed from 63 to 77 days of development. In *A. pisum*, at temperatures around 10 and 16 °C, katatrepsis was observed a little earlier [32]. 

The process of creating embryo’s cuticle is unclear. The main enzyme for chitin-synthesis pathway is chitin synthase (CHS). Most insects have two chitin synthase which are encoded by genes CHS1 and CHS2 [37,38]. To date, characteristic insect chitin synthases have been noted from at least 15 separate holometabolous insects, but only in three hemimetabolous [38,39,40,41,42]. *CHS1* is mainly responsible for the exoskeleton structures and is important for production of chitin required for cuticle and tracheae. This gene also plays an important role during insect development [40]. *CHS2* is expressed in the midgut and is required for production of chitin in the PM (peritrophic membrane) of insect midgut [38]. In aphids, there is only one of these genes *CHS*. Its size in *A.glycines* is similar to the *CHS1* cDNA in other insects [38]. It is known that the cuticle of *A. pisum* completely covers the body of the aphid from the 84th day of development [32]. However, our research showed that the cuticle of *Cinara* embryos are visible since the 77th day of development. At the front end of the body, a delicate but well-visible egg burster was observed, which later became stronger and stronger in the following days. The egg burster function is to help with the hatching process by visible serrated spines. From 84 days, short hair covering the cuticle was also observed. Future studies on CHS gene expression in the early stages of aphid development would be advantageous to understand when the process of creating a cuticle begins.

Earlier research has indicated that the diapause process should consist of a period of rest, referred to as the “resting stage” [27]. The process of “diapause development” described earlier, referred rather to changes at the physiological level observed during the “resting stage”, not morphological ones [11,14]. The recent research suggests that one of the hallmarks of insect diapauses is cell cycle arrest in tissues, as observed in larval of fly *Chymomyza costata*, silkworm *Bombyx mori,* and fleshfly *Sarcophaga crassipalpis* [12]. Cell cycle arrest in the G0/G1 or G2 phase was observed for these insects. The essence of the definition of diapause, however, is the predictable seasonal response to environmental stress, genetically programmed, under hormonal control, which lasts for a certain period of time. Cell cycle arrest observed in some species may not be an obligatory feature and may be due to differences between Holometabola and Hemimetabola insects. Many Holometabola species, for example some mosquito species, are additionally able to go to quiescence, as a way to quickly respond to acyclic environmental stress (disturbances) [9]. Insects that have larvae, pupae, and adults in their development and can produce several generations in a season are forced to protect each stage of their development against environmental disturbances. However, holocyclic aphids have a different life strategy, because their eggs are laid only once a season before the regular environmental stress (winter). Thus, it seems that embryos of *C. cupressi* and *C. juniperi* undergo diapause without a “resting stage”. Perhaps, diapause in many other insects may also involve a ‘slowing’, not a cessation of morphological development, but few studies have been conducted so far. A few examples of continued morphological development during diapause have been observed in embryos of the orthopteran, caterpillars, and only for one species of aphids *A. pisum* [14,32,43]. Consequently, diapause could be considered an extreme regulation of developmental rate, rather than a shutting down of morphological development; and the mechanisms that control diapause in *A. pisum* may therefore be the same as those that control the rate of morphological development at other stages of development [32]. Many insects that undergo diapauses in winter grow only at the beginning of winter and post-diapause period. Our research clearly shows that the *Cinara* aphids develop during the entire winter season. However, the major process of development is completed early, to the end of anatrepsis and then the embryo undergoes a type of diapause, with slow growth. There is a probability that the aphids develop at different development rates during and after diapause. There was no obvious difference in development of *A. pisum* during diapause periods, in contrast to species of *Cinara*. The developmental rate of the *Cinara* aphids was more intensive than *A. pisum*, so we suggest that the phenomena previously observed in *A. pisum* is not unique and also applies to the *Cinara* species. The increase in body length of *Cinara* species was greater than *A. pisum* during diapause (until the beginning of katatrepsis). While the length of the body of embryo of the *C. juniperi* grew 21% and *C. cupressi* 29%, the body of *A. pisum* grew only about 13% (based on the rough data available in [32]). The increase in body length after katatrepsis was clearly greater in *C. cupressi* and was about 40%, and similarly in *C. juniperi* and *A. pisum*, where it was 25% and about 26%, respectively. We didn’t observe any morphological anomalies in embryos. The malformed phenotypes of aphids were found in the case transfer aphids from 0–4 °C to 16 °C on day 49 [32]. 

The duration of diapause is programmed genetically, but also by the conditions in the course of diapause induction [44,45]. A number of authors suggested a role for insulin, which regulates growth in other stages of development in *Drosophila* [18,46,47] The second class of genes that may be involved in diapause regulation are the heat-shock proteins. They are also involved in cell cycle arrest, but they can also play an important role in the regulation of diapause [15]. Heat-shock proteins are related to stress responses and protect the cell against thermal stress. It also indicates the possibility of potentially using HSP genes as bioindicators of diapause and thermal stress in moth *Grapholita molesta* [48]. HSPs also played an important role in temperature tolerance of aphid *C*. *pinitabulaeformis* (Zhang and Zhang, 1989) [49]. In general, HSPs are up-regulated by stress conditions [50] and their transcription and translation are quickly induced as a response to stress [51,52], so the authors suggested the relations of HSPs with diapause process of insects [53,54,55]. Because the increased stress tolerance is a conserved feature of diapauses, proteins promoting stress tolerance, are up-regulated in diapausing insects [55,56]. HSPs may play the role of “inhibitory factors”, which functions as a chaperone, to inhibit abnormal protein folding in harsh environmental conditions and regulating the diapause process. Our research indicates that during diapauses, the successive stages of aphids differ from each other in their rate of development. The changes in activity of HSPs may be crucial in understanding diapause regulation. Further examinations explaining the role heat-shock proteins in aphid’s embryos during diapause period, are necessary.

## 5. Conclusions

Our results indicated that the “resting stage” does not occur in embryos of two aphid species belonging to the *Cinara* genus. Their embryos grow and develop throughout the duration of the winter diapause. It seems that this feature is conducive to the development of embryos in the egg, which may be one of the features of aphid wintering.
